# Detection of pre-existing immunity to bacterial Cas9 proteins in people with cystic fibrosis

**DOI:** 10.1093/immhor/vlaf041

**Published:** 2025-09-17

**Authors:** Gregory Serpa, Qiaoke Gong, Mithu De, Pranav S J B Rana, Christopher P Montgomery, Daniel J Wozniak, Matthew E Long, Emily A Hemann

**Affiliations:** Department of Internal Medicine, Division of Pulmonary, Critical Care, and Sleep Medicine, College of Medicine, The Ohio State University Wexner Medical Center, Columbus, OH, United States; Department of Microbial Infection and Immunity, College of Medicine, The Ohio State University Wexner Medical Center, Columbus, OH, United States; Department of Microbial Infection and Immunity, College of Medicine, The Ohio State University Wexner Medical Center, Columbus, OH, United States; Department of Internal Medicine, Division of Pulmonary, Critical Care, and Sleep Medicine, College of Medicine, The Ohio State University Wexner Medical Center, Columbus, OH, United States; Department of Microbial Infection and Immunity, College of Medicine, The Ohio State University Wexner Medical Center, Columbus, OH, United States; Center for Microbe and Immunity Research, Abigail Wexner Research Institute at Nationwide Children’s Hospital, Columbus, OH, United States; Department of Pediatrics, College of Medicine, The Ohio State University Wexner Medical Center, Columbus, OH, United States; Department of Microbial Infection and Immunity, College of Medicine, The Ohio State University Wexner Medical Center, Columbus, OH, United States; Department of Internal Medicine, Division of Pulmonary, Critical Care, and Sleep Medicine, College of Medicine, The Ohio State University Wexner Medical Center, Columbus, OH, United States; Department of Microbial Infection and Immunity, College of Medicine, The Ohio State University Wexner Medical Center, Columbus, OH, United States; Dorothy M. Davis Heart and Lung Research Institute, College of Medicine, The Ohio State University Wexner Medical Center, Columbus, OH, United States; Department of Microbial Infection and Immunity, College of Medicine, The Ohio State University Wexner Medical Center, Columbus, OH, United States; Dorothy M. Davis Heart and Lung Research Institute, College of Medicine, The Ohio State University Wexner Medical Center, Columbus, OH, United States

**Keywords:** antibody, Cas9, cystic fibrosis, T cell

## Abstract

Cystic fibrosis (CF) is caused by homozygous mutations in the cystic fibrosis transmembrane conductance regulator (*CFTR*) gene, resulting in multi-organ dysfunction and decreased lifespan and quality of life. A durable cure for CF will likely require a gene therapy approach to correct CFTR. Rapid advancements in genome editing technologies, including CRISPR/Cas9, have already resulted in Food and Drug Administration (FDA) approval for cell-based gene editing therapies, providing new therapeutic avenues for many rare diseases. However, immune responses to gene therapy delivery vectors and editing tools remain a challenge, especially for strategies targeting complex *in vivo* tissues such as the lung. Previous findings in non-CF healthy individuals reported pre-existing antibody and T cell responses to recombinant Cas9 proteins, suggesting potential additional obstacles for incorporation of clustered regularly interspaced short palindromic repeats (CRISPR)/Cas9 technologies in gene therapies. To determine whether pre-existing immunity to Cas9 from *S. aureus* or *S. pyogenes* was present or augmented in people with CF, anti-Cas9 IgG levels and Cas9-specific T cell responses were determined from peripheral blood samples of people with CF and non-CF healthy controls. Overall, non-CF control and CF samples displayed evidence of pre-existing antibody and T cell responses to both *S. aureus* and *S. pyogenes* Cas9, although there were no significant differences between these populations. However, we observed global changes in CF activation of Th1 and CD8 T cell responses as measured by interferon γ (IFN-γ) and tumor necrosis factor (TNF) that warrant further investigation and mechanistic understanding as this finding has implications not only for CRISPR/Cas9 gene therapy for people with CF but also for protection against infectious disease.

## Introduction

Cystic fibrosis (CF) is a genetic disease hallmarked by a defective cystic fibrosis transmembrane conductance regulator (CFTR) protein that results in severe multi-organ disease manifestations with particularly damaging effects for the lung. While new highly effective CFTR modulator therapies (HEMT) have resulted in rapid reductions of disease burdens for people with CF, there remains a significant population of people with CF who cannot benefit from HEMT. Globally, there are additional major barriers restricting access to CF standards of care, including HEMT. Although HEMT is rightfully considered a breakthrough achievement to treat the underlying cause of CF, it is not a permanent cure for CF. Therefore, development of approaches to correct the underlying *CFTR* genetic mutations and restore CFTR function is a long-term goal of CF research.^1^^,^^2^ The discovery of the clustered regularly interspaced short palindromic repeats (CRISPR)/Cas9 system has resulted in new molecular tools to conduct precision gene editing and has rapidly transformed approaches to gene therapy.^3^ A recent example for successful application of these technologies is the first Food and Drug Administration (FDA) approved therapy that utilizes CRISPR/Cas9 genome editing technology in CD34^+^ hematopoietic stem cells to treat transfusion-dependent B-thalassemia, a form of sickle cell disease.^4^ This represents a major milestone in genetic therapies and demonstrates the potential utility of CRISPR/Cas9 genome editing approaches.

Many of the initial CRISPR/Cas9 genome editing tools utilized Cas9 proteins derived from bacterial species such as *Staphylococcus aureus* or *Streptococcus pyogenes,*^5^^,^^6^ and the FDA approved treatment utilizes a synthetic guide RNA and a *S. pyogenes* Cas9 endonuclease.^7^^,^^8^ As many people have either been colonized or exposed to *S. aureus* and *S. pyogenes*, it could be predicted that humans may harbor some level of adaptive immune responses to Cas9 proteins that could specifically target and eliminate cells subjected to and expressing this component of a gene editing vector. Indeed, recent discoveries showed that heathy adults harbored both antibody and cellular immune responses against recombinant Cas9 proteins.^9^^,^^10^ Furthermore, many people with CF have recurrent and chronic infections that start early in life,^11^ and *S. aureus* is one pathogen commonly associated with these infections. Therefore, we hypothesized that people with CF may have enhanced pre-existing immunity to Cas9 proteins compared to a non-CF population, which could function as a barrier to effective utilization of CRISPR/Cas9 therapies in CF. To test this hypothesis, we used ELISA based methods to evaluate the levels of anti-Cas9 immunoglobulin G (IgG) and assessed T cell naïve, memory, and regulatory population levels in addition to cytotoxic molecule expression and cytokine production following Cas9 peptide stimulations of peripheral blood mononuclear cells (PBMC).

Here, we present results from a single-center study that evaluated the presence of pre-existing adaptive immune responses to *S. aureus* or *S. pyogenes* Cas9 in healthy controls and people with CF. Results from this study demonstrate similar IgG responses between CF and non-CF control samples, which supports the presence of pre-existing immunity to Cas9 proteins. We identified similar CD4 and CD8 T cell production of interferon γ (IFN-γ) and tumor necrosis factor (TNF) in CF and non-CF control cells when stimulated with peptide pools from *S. aureus* or *S. pyogenes* Cas9. Additionally, we observed similar frequencies of granzyme B^+^ and CD107a^+^ CD4 and CD8 T cells, suggesting similar cytotoxic capabilities between CF and non-CF control T cells. To our knowledge, these results are the first demonstration of pre-existing immunity to Cas9 proteins in people with CF. These findings suggest inclusion of methods to evaluate pre-existing and development of immune responses to Cas9 proteins in future human gene therapy approaches in CF that utilize CRISPR-based gene editing technologies. Intriguingly, while no difference in the ability of CD4 and CD8 T cells to produce IFN-γ and TNF following Cas9 or positive control CEF (cytomegalovirus [CMV], Epstein-Barr virus [EBV], influenza virus) peptides was observed, there was a significant reduction in the frequency of IFN-γ^+^/TNF^+^ CD4 and CD8 T cells in CF compared to non-CF controls when stimulated with phorbol 12-myristate 13-acetate and ionomycin (PMA/I). FoxP3^+^ CD4 T regulatory cells were also significantly reduced in CF compared to non-CF control samples, confirming previously reported findings in the context of bacterial infection in individuals with CF.^12^^,^^13^ Together, these results demonstrate that there are no alterations in the Cas9 specific response in people with CF, however there are broad defects in the ability of CF CD4 and CD8 T cells to produce CD8/Th1 effector cytokines that warrant further study.

## Materials and methods

### Human blood and PBMC cryopreservation

Anti-coagulated peripheral blood was collected by venous puncture and plasma samples collected following centrifugation at 16,000×*g* for 10 min at 4 °C in Microtainer^®^ Blood Collection Tubes (BD catalog no. 365967). Blood from non-CF and people with CF was provided as de-identified biospecimens from the Cure CF Columbus Research Development Program Translational and Data Core under the NCH IRB and OSU IRB 2020H0399. Several additional non-CF samples were provided from OSU IRB 2014H0154. Plasma samples were stored at -80°C until use for ELISA endpoint measurements. PBMC were isolated by gradient centrifugation utilizing Ficoll-Paque Plus (Sigma, catalog no. GE17-1440-02) according to manufacturer’s instructions. Here, 1 × 10^7^ total cells were resuspended in 90% HI-FBS + 10% DMSO and cryopreserved for storage in liquid nitrogen.

### ELISA reagents and protocol

Samples were processed in batches, where n = 11 samples were thawed and used at the same time for assessment in four endpoint ELISAs for human serum albumin (HSA), tetanus toxoid, *S. aureus* Cas9, or *S. pyogenes* Cas9. ELISA methods were adapted from those previously reported.^9^  *S. aureus* Cas9 (Sa Cas9) SaCas9 without a nuclear localization signal (No NLS) and *S. pyogenes* Cas9 (Sp Cas9) SpCas9 V3 No NLS were obtained from IDT. HSA was obtained from Sigma Aldrich (catalog no. SRP6182) and inactivated tetanus toxoid was obtained from Cellero (catalog no. 1002). Nunc Maxisorp ELISA plates (Fisher catalog no. 12-565-135) were coated overnight at 4°C by dilution into 100 μl of carbonate-bicarbonate coating buffer (Sigma Aldrich, catalog no. C3041-50CAP) to 0.5 μg of antigen per well for each of the recombinant Cas9 proteins, tetanus toxoid, or HSA. Following overnight coating, wells were washed 3 times with Tris buffered saline with Tween-20 (Sigma Aldrich, catalog no. T9039-10PK), and plates were incubated with 200 μl blocking solution for 1 hour at room temperature. Blocker BSA (10%) from ThermoFisher (catalog no. 37525) was diluted to 1% in phosphate buffered saline (PBS) for the blocking solution. Sample dilutions were performed in blocking solution (1% BSA in PBS) and were plated in technical duplicates. Plates were washed three times following blocking and 100 μl of samples were added to each well and incubated at room temperature for 2 hours. Plates were washed 3 times, followed by incubation with 200 μl of diluted secondary antibody for 1 hr at room temperature. The goat polyclonal, goat anti-human IgG Fc secondary antibody (R&D Systems, catalog no. NB7449) was diluted 1:100,000 in 1% BSA. Plates were washed an additional three times and 100 μl of room temperature development reagent was added to each well. Plates were incubated at room temperature protected from light; a timer was used to precisely track the development time of each plate, and 50 μl of 1 N sulfuric acid was added after 10 min to end the reactions. The absorbance at 450 nm was quantified using a Spectramax ID3 plate reader.

### PBMC stimulation and flow cytometry

Peptide stimulations were performed as previously described.^14^ Frozen PBMC were rapid thawed in complete RPMI containing 2 μl benzonase (Sigma, catalog no. 706643), centrifuged at 350×*g* for 10 min at 4 °C, and resuspended in a 96-well U-bottom plate (5 × 10^5^ cells/well). Media, defined *S. aureus*^15^^,^^16^ or *S. pyogenes*^17^ peptide pools (Table 1), or Peptivator CEF MHC Class I Plus positive control peptide pool (Miltenyi, catalog no. 130-098-426) was added to cells with brefeldin A (eBioscience, catalog no. 00-4506-51), recombinant human interleukin (IL)-2 (Sigma, catalog no. 11011456001) and anti-human CD107a AF700 (BD, catalog no. 561340) were added to wells and incubated at 37 °C for 24 hr. Cell stimulation cocktail containing PMA/I (Invitrogen, catalog no. 00-4970) was added to unstimulated wells for the final 6 hr of the incubation period. Following incubation, cells were labeled with Live/Dead Fixable Blue Dead Cell Stain Kit (Invitrogen, catalog no. L34962) according to manufacturer instructions. Samples were then blocked with Human TrueStain FcX (BioLegend, catalog no. 422302) for 10 min at room temperature and subsequently surface stained with the following fluorescently conjugated antibodies for 30 min on ice: anti-human CD3 APC-H7 (BD, catalog no. 560176), anti-human CD4 BUV496 (BD, catalog no. 612936, anti-human CD8 BUV805 (BD, catalog no. 612889), anti-human CD45R0 BV570 (BioLegend, catalog no. 304226), anti-human CD45RA AF647 (BioLegend, catalog no. 305154). Samples were fixed and permeabilized with BioLegend True-Nuclear Transcription Factor Buffer Kit (catalog no. 424401) according to manufacturer’s instructions. Following permeabilization, samples were blocked as described above and stained with the following antibodies specific for intracellular antigens for 40 min on ice: anti-human FoxP3 Pacific Blue (BioLegend, catalog no. 320116), anti-human IFN-γ BB700 (BD, catalog no. 566394), anti-human TNF BV750 (BD, catalog no. 566359) anti-human granzyme B FITC (BioLegend, catalog no. 515403). Samples were washed and resuspended in PBS to run on a Cytek Aurora flow cytometer. Samples were analyzed using FlowJo (BD). Upstream of representative flow panels and populations shown on graphs samples were gated on viable CD3^+^CD4^+^CD8^−^ cells (CD4 T cells) or viable CD3^+^CD8^+^CD4^−^ cells (CD8 T cells).

**Table 1.** *Staphylococcus aureus* and *Streptococcus pyogenes* peptides utilized for T cell stimulation.

	Peptide position	Peptide sequence	Citation
Sa Pool 1	Sa Cas9_849-869	DEKNPLYKYYEETGNYLTKYS	^15^
	Sa Cas9_862-876	GNYLTKYSKKDNGPV	^15^
	Sa Cas9_918-935	LDNGVYKFVTVKNLDVIK	^15^
	Sa Cas9_936-951	KENYYEVNSKCYEEAK	^15^
	Sa Cas9_956-973	ISNQAEFIASFYNNDLIK	^15^
	Sa Cas9_964-972	FIASFYNND	^16^
Sp Pool 1	Sp Cas9_511-529	VLPKHSLLYEYFTVYNELT	^17^
	Sp Cas9_615-623	ILEDIVLTL	^17^
	Sp Cas9_615-629	ILEDIVLTLTLFEDR	^17^
Sp Pool 2	Sp Cas9_836-852	RLSDYDVAAIVPQSFLK	^17^
	Sp Cas9_883-898	MKNYWRQLLNAKLITQ	^17^
	Sp Cas9_988-997	YLNAVVGTAL	^17^
	Sp Cas9_1036-1051	ATAKYFFYSNIMNFFK	^17^

### Data analysis and statistics

Statistical analyses and calculations of the area under the curve were performed in GraphPad Prism Version 10.4.1. The specific statistical tests used are listed in individual figure legends. GraphPad Prism Version 10.4.1 was used for all statistical analysis.

## Results

### Blood IgG specific for Cas9 proteins is prevalent and equivalent in non-CF control and CF samples

**Figure 1.**
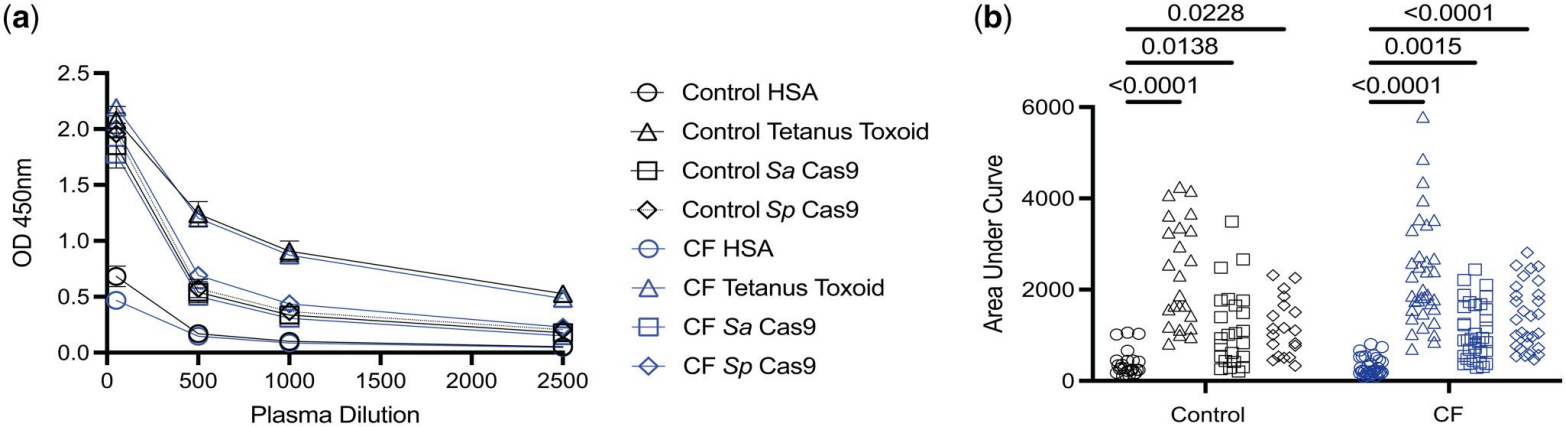
Detection of antibodies in blood of non-CF healthy controls and people with cystic fibrosis. (A) Mean ± SEM of ELISA measurements from each group (HSA [n = 28 non-CF control, n = 34 CF], tetanus toxoid [n = 28 non-CF control, n = 34 CF], *S. aureus* Cas9 [Sa Cas9] [n = 28 non-CF control, n = 34 CF], *S. pyogenes* [Sp Cas9] [n = 19 non-CF control, n = 29 CF]) demonstrate OD450 nm endpoint values across sample dilutions of 1:50, 1:500, 1:1000, and 1:2500. (B) The area under the curve from the cumulative data obtained from all ELISA measurements for HSA (n = 28 non-CF control, n = 34 CF), tetanus toxoid (n = 28 non-CF control, n = 34 CF), Sa Cas9 (n = 28 non-CF control, n = 34 CF), Sp Cas9 (n = 19 non-CF control, n = 29 CF). Statistical comparisons were performed by two-way ANOVA with Tukey’s multiple comparison test; there were no statistically significant differences comparing non-CF control or CF for each individual ELISA.

To evaluate whether antibodies to recombinant Cas9 proteins exist in people with CF, an ELISA-based method was adapted for this study.^18^ Samples were obtained from non-CF healthy control and people with CF. We obtained 24 non-CF control samples from individuals that were 79% female with a median age of 32.5 yr. Thirty-five samples were collected from individuals with CF that were 57% female with a median age of 24 yr. ELISAs were performed to detect relative levels of anti-*S. aureus* Cas9 (Sa Cas9) and anti-*S. pyogenes* Cas9 (Sp Cas9) IgG utilizing anti-HSA IgG as a negative control, and anti-tetanus toxoid IgG as a positive control.^9^ Based on the dilution curves, IgG levels between non-CF control and CF are similar for each antibody specificity (Fig. 1A). Calculation of the area under the curve (AUC) to quantify antigen-specific IgG levels (Fig. 1B) revealed statistically significant increases in the levels of tetanus toxoid, Sa Cas9, and Sp Cas9 compared to HSA for non-CF control and CF, although there were no significant differences when comparing non-CF control or CF responses to either Cas9 or the tetanus toxoid positive control.

We performed an additional more stringent analysis by assessing whether individual samples had positive antibody responses for Cas9 or tetanus toxoid defined by an AUC greater than 3 standard deviations above the mean AUC for the HSA negative control (from data shown in Fig. 1B). As expected, antibody positivity to the positive control tetanus toxoid was high, with 89% of all samples having positive tetanus toxoid antibody responses, with no difference between non-CF control (87%) and CF samples (91%). We detected positive antibody responses to *S. aureus* (Sa) Cas9 in 58% of non-CF control and 57% of CF samples, while 52% of non-CF control and 75% of CF samples had positive antibody responses to *S. pyogenes* (Sp) Cas9. Overall, these results suggest that the prevalence of pre-existing antibody responses to Sa Cas9 and Sp Cas9 is relatively high in both non-CF and CF populations, consistent with previous findings reported in a cohort of healthy humans.^9^

### Cas9-specific cytotoxic molecules and IFN-γ/TNF production by CD4 and CD8 cells are equivalent in non-CF control and CF samples

**Figure 2.**
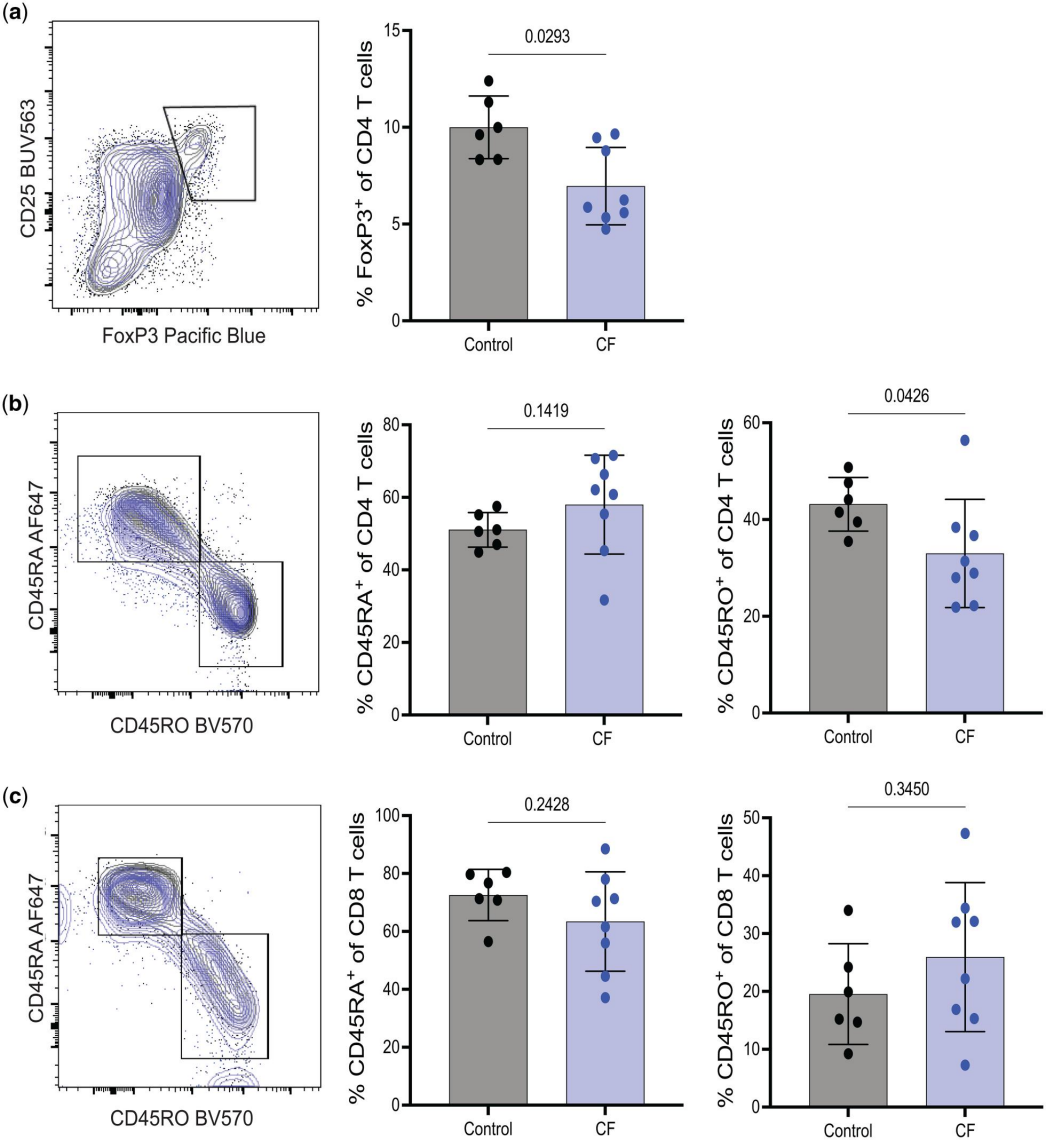
People with CF have decreased frequency of FoxP3^+^ CD4 Treg, but equivalent CD4 and CD8 T cell naïve and memory cell frequency compared to non-CF healthy controls. PBMCs were thawed and incubated in complete media containing IL-2 and brefeldin A for 24 hr, with PMA/I added for the last 6 hr. Cells were stained to identify (A) FoxP3^+^ CD4 T cells as well as CD45RA (naïve) and CD45RO (memory) CD4 (B) and CD8 (C) T cell populations by flow cytometry. Representative panels show gating of populations with healthy controls (black) and CF (blue) overlayed. Data points represent cells from an individual donor (n = 6 non-CF controls and n = 8 CF); bars represent the mean ± SD. Statistical comparisons were performed using an unpaired Student *t*-test.

While antibodies are indicative of pre-existing immunity that may correlate to immune responses targeting Cas9 proteins or Cas9 gene-edited cells, cytotoxic CD8 and CD4 T cell responses could result in the functional targeting of gene-edited cells expressing Cas9 via MHCI and MCHII, respectively. Therefore, we next investigated T cell immunity in a small cohort of samples from non-CF controls (n = 8, median age 39.5 yr, 83% female) and CF (n = 6, median age 31.5 yr, 63% female). PBMC were stimulated with a number of previously identified *S. aureus* and *S. pyogenes* immunostimulatory Cas9 peptides, or CEF or PMA and ionomycin (PMA/I) as positive controls to determine if T cells from people with CF have altered responses specific for *S. aureus* or *S. pyogenes* Cas9 peptides compared to non-CF controls.^15–17^ Following 24 hr of stimulation, flow cytometry analyses were performed to quantify the frequency of naive, memory, and regulatory T cells (Treg). Although T cell immunity in CF has not been widely reported in the literature, FoxP3^+^ CD4 Treg have been previously described to be significantly reduced in CF.^19^^,^^20^ Specifically, reduced Treg responses in CF with chronic *P. aeruginosa* infection have been observed.^13^ While the Treg frequency was significantly increased in vitro following HEMT treatment,^12^ whether these levels are rescued to levels equivalent to non-CF control individuals is unknown. Despite the small cohort in our study, we confirmed that the frequency of peripheral Foxp3^+^ CD4 T cells was significantly reduced in CF compared to controls (Fig. 2A). We also determined that the frequencies of CD45RA^+^ (generally naïve) and CD45RO^+^ (generally memory) CD4 (Fig. 2B) and CD8 (Fig. 2C) T cells in the peripheral blood revealed no dramatic changes between non-CF control and CF samples, suggesting the distribution of naïve and memory T cell populations in the peripheral blood is unaltered in people with CF.

**Figure 3.**
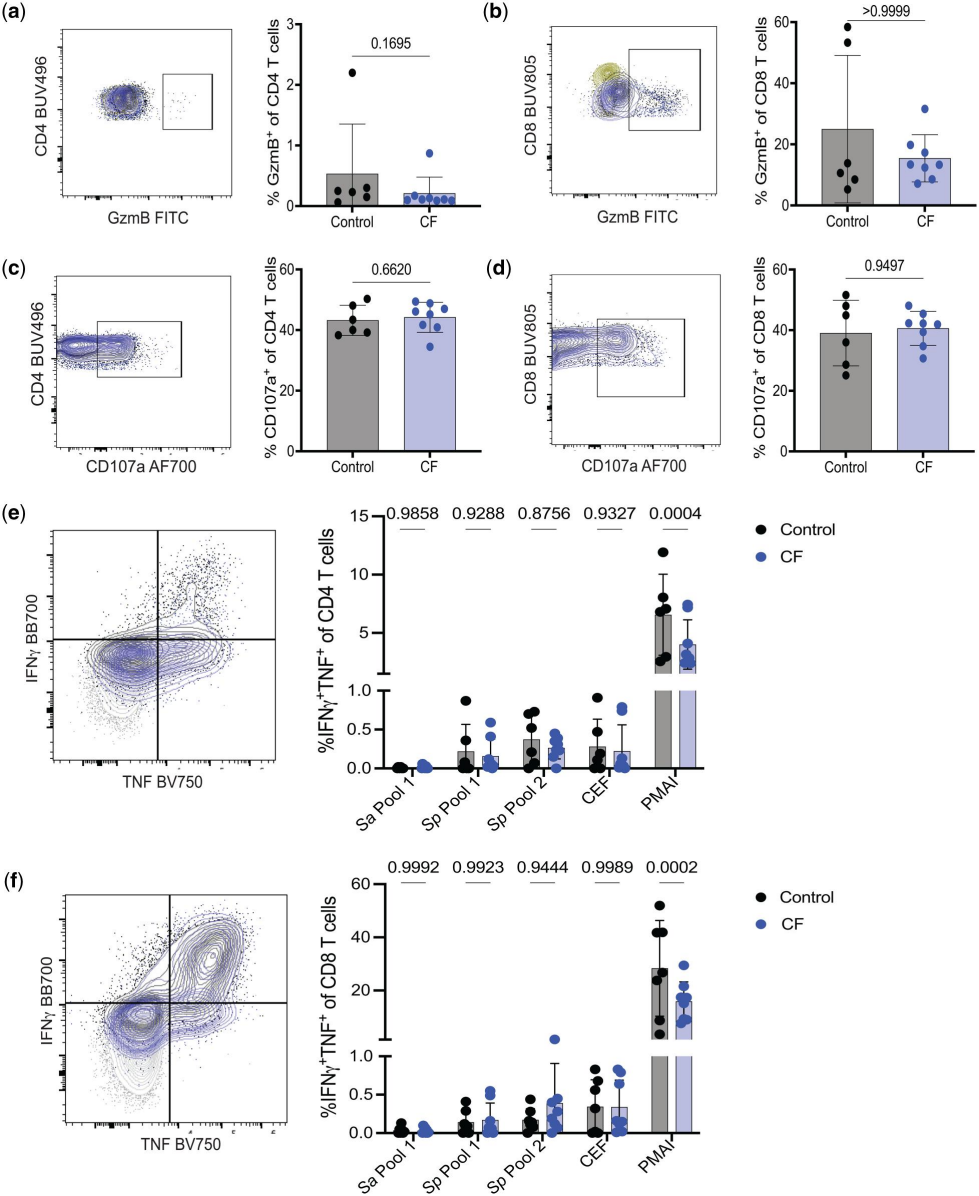
People with CF have equivalent responses to Cas9 peptides but a reduced total capacity for IFN-γ/TNF production compared to non-CF healthy controls. (A–D) PBMCs were thawed and incubated in complete media containing IL-2, brefeldin A, and anti-CD107a for 24 h, with PMA/I added for the last 6 hr. Cells were stained to identify the frequency of granzyme B^+^ CD4 (A) and CD8 (B) T cells as well as the frequency of CD107a^+^ CD4 (C) and CD8 (C) T cells. Representative flow plots show gating for populations of healthy controls (black) and CF (blue) compared to isotype control (yellow). (E, F) PBMCs were thawed and incubated in complete media containing IL-2 and brefeldin A with Sp, Sa, or CEF peptides in Table 1 for 24 hr, or PMA/I added to the complete media for the last 6 hr. Cells were stained to identify the frequency of IFN-γ^+^/TNF^+^ CD4 (E) and CD8 (F) T cells. Representative flow plots show gating for populations of healthy controls (black) and CF (blue) stimulated with PMA/I compared to baseline media incubation (grey). Data points represent cells from an individual donor from n = 6 non-CF controls and n = 8 CF and bars represent the mean ± SD. Statistical comparisons were performed using an unpaired Student *t*-test (A–D) or two-way ANOVA with Tukey’s multiple comparison test (E and F).

To determine whether the cytotoxic ability of CD4 and CD8 T cell responses was altered in CF, we measured the intracellular expression of granzyme B (gzmB) and found no significant differences in the percentage of gzmB^+^ CD4 (Fig. 3A) or CD8 (Fig. 3B) T cells between non-CF and CF samples. Additionally, we included fluorescently labeled anti-CD107a antibody during a 6 hr stimulation with PMA/I in the presence of IL-2 and brefeldin A to serve as a proxy measure of cytotoxic degranulation, as the antibody would bind CD107a expressed on the surface of cells during degranulation for analysis by flow cytometry.^21^ The frequency of CD107a^+^ CD4 (Fig. 3C) or CD8 (Fig. 3D) T cells was not altered in CF compared to non-CF controls. Together, these findings demonstrate no alteration in cytotoxic molecule expression in CF compared to non-CF control T cells.

As anticipated based on previous studies identifying the peptides utilized (Table 1), we detected low frequencies of CD4 T cells producing IFN-γ and TNF following stimulations (Fig. 3E), although responses specific to the *S. aureus* (Sa) peptide pool were largely undetectable. However, CD4 IFN-γ/TNF production in response to both *S. pyogenes* (Sp) peptide pools was similar to the CEF positive control peptide pool, and responses were equivalent between control samples and CF (Fig. 3E). Similar results were observed when we assessed IFN-γ and TNF production by CD8 T cells following stimulation with Sa, Sp, or CEF peptide pools (Fig. 3F). In summary, these results indicate that pre-existing effector CD8 and Th1 CD4 T cell responses specific to Cas9 can be detected and are equivalent between non-CF controls and CF samples. Critically, these finding suggest there may be no additional or enhanced barrier to development of gene therapy treatments in individuals with CF compared to non-CF populations due to similar levels pre-existing Cas9-specific antibody and T cell responses.

Although we observed no significant differences in the ability of non-CF control or CF CD4 or CD8 T cells to produce IFN-γ/TNF in response to *S. pyogenes*, *S. aureus*, or CEF peptides, we noted a striking and significant reduction in the frequency of both CD4 (Fig. 3E) and CD8 (Fig. 3F) CF T cells producing IFN-γ/TNF following stimulation with PMA/I compared to non-CF controls. This suggests that while the antigen-specific responses investigated were equivalent between non-CF control and CF samples, the maximal effector response is specifically limited in CF. Whether these CF T cells responses are blunted in response to infection or health status of the blood donors or other stimuli remains unknown. In summary, while this study indicates no additional risks for adaptive immune-mediated elimination of Cas9 targeted cells that would hamper gene targeting efforts in CF, we have identified a previously unreported defect in the ability of Th1 and CD8 T cells to produce IFN-γ/TNF in response to PMA/I.

## Discussion

Here, we performed the first assessment to our knowledge of pre-existing immunity to Cas9 proteins in CF. Results from this study revealed that consistent with non-CF individuals,^9^^,^^10^^,^^15^ individuals with CF also have pre-existing antibody responses to Cas9. While recent success utilizing *ex vivo* correction of Sickle cell disease is one example of encouraging success for gene therapy to cure human disease, this procedure requires myeloablation prior to deliver of edited cells that eliminates these potential pre-existing immune responses that may target edited cells.^22^ Pre-existing immunity to CRISPR/Cas9 technologies may severely impact the success of any attempt to correct human *CFTR* mutations as these pre-existing Cas9-specific responses would be maintained following gene editing. Therefore, the identification of pre-existing antibody and T cell immunity in individuals may allow for development of modified Cas9 proteins to avoid these responses. In this regard, pre-existing immunity has already been the target of several studies that have attempted to silence these responses to improve gene targeting effectiveness.^16^^,^^17^ Overall, we believe results from this study may serve as a roadmap to incorporate these data into the efforts to find a permanent cure for CF.

In the current study, Cas9 peptide stimulations confirm the ability of PBMC from people with CF to respond even though no significant differences were observed compared to healthy non-CF control samples. Overall, while these results are consistent with the presence of T cell responses specific to Cas9 proteins reported in healthy controls,^10^ one limitation of the current study is the small cohort size and lack of HLA phenotyping for the PBMC stimulations. This could contribute to some variability and potentially lower T cell (or PBMC) responses as we used peptide pools capable of stimulating MHCI and MHCII rather than screening to identify specific peptides associated with responses for specific HLA MHCI or MHCII types. While future studies utilizing a larger cohort of individuals with defined HLA haplotypes would provide a more robust assessment of which *S. aureus* and *S. pyogenes* epitopes individuals are likely to be responsive to, our data demonstrate no significant changes in the frequency of CD4 and CD8 T cells expressing IFN-γ and TNF. Our study additionally demonstrated that the frequency of CD4 and CD8 T cells expressing CD107a and granzyme B were also equivalent between non-CF control and CF samples. One limitation of the current experimental design is that the data only provide a surrogate of T cell function and did not directly assess the T cell mediated killing of target cells, which could be incorporated into future studies. Together, these results suggest there is no significant alteration in the cytotoxic T cell responses in people with CF that could potentially target cells that have been treated with CRISPR/Cas9 for CFTR mutation correction. However, the high prevalence of Cas9-specific antibody responses suggest monitoring of these responses in conjunction with effectiveness of CRISPR/Cas9 gene correction therapeutics is warranted.

While no significant alterations in Cas9-specific T cell responses were observed in this study, we did note global alteration in CF CD8 T cell cytokine production after stimulation with PMA/I. Following PMA/I stimulation, IFN-γ/TNF producing CD4 and CD8 T cell frequencies were significantly reduced in CF compared to non-CF controls. This suggests specific pathways downstream of PMA/I activation (including NFAT and NF-kB) may be defective compared to peptide-dependent activation by antigen presenting cells. While a recent study identified airway fluid and neutrophils from CF to inhibit proliferation of T cell responses,^23^ these were not included in the experimental methods for our current study. Finally, while skewing toward Th2 CD4 T cell gene expression and limited Th1 gene expression has previously been shown to be associated with exacerbation and bacterial colonization,^24^ the underlying mechanisms responsible for these phenotypes are not well understood and there remains a lack of specific investigation of potential alterations of the function of Th1 or CD8 T cell responses in CF. Future studies with larger cohorts will be critical to determine the full spectrum of defects in CD8 T cell responses in CF.

There are several important limitations of the current study. First, our analyses include assessment of CF samples from a single clinical research center and therefore require validation in additional cohorts. Second, we do not have information on the infection history regarding *S. aureus* and *S. pyogenes* in the sample cohort, limiting the ability to perform an analysis on whether infection status correlates with cellular or antibody responses. In summary, these findings have potentially important implications regarding protective immune response to pathogens in people with CF. Additional studies investigating adaptive immunity in CF will be important to understand CF disease in people with CF in the context of both prolonged life expectancy due to HEMTs as well as the emergence of potential gene editing technologies.

## Data Availability

Raw data are available upon request.
